# Role of Choline Deficiency in the Fatty Liver Phenotype of Mice Fed a Low Protein, Very Low Carbohydrate Ketogenic Diet

**DOI:** 10.1371/journal.pone.0074806

**Published:** 2013-08-29

**Authors:** Rebecca C. Schugar, Xiaojing Huang, Ashley R. Moll, Elizabeth M. Brunt, Peter A. Crawford

**Affiliations:** 1 Department of Medicine, Center for Cardiovascular Research, Washington University, St. Louis, Missouri, United States of America; 2 Department of Pathology and Immunology, Washington University, St. Louis, Missouri, United States of America; 3 Department of Genetics, Washington University, St. Louis, Missouri, United States of America; State University of Rio de Janeiro, Biomedical Center, Institute of Biology, Brazil

## Abstract

Though widely employed for clinical intervention in obesity, metabolic syndrome, seizure disorders and other neurodegenerative diseases, the mechanisms through which low carbohydrate ketogenic diets exert their ameliorative effects still remain to be elucidated. Rodent models have been used to identify the metabolic and physiologic alterations provoked by ketogenic diets. A commonly used rodent ketogenic diet (Bio-Serv F3666) that is very high in fat (~94% kcal), very low in carbohydrate (~1% kcal), low in protein (~5% kcal), and choline restricted (~300 mg/kg) provokes robust ketosis and weight loss in mice, but through unknown mechanisms, also causes significant hepatic steatosis, inflammation, and cellular injury. To understand the independent and synergistic roles of protein restriction and choline deficiency on the pleiotropic effects of rodent ketogenic diets, we studied four custom diets that differ only in protein (5% kcal vs. 10% kcal) and choline contents (300 mg/kg vs. 5 g/kg). C57BL/6J mice maintained on the two 5% kcal protein diets induced the most significant ketoses, which was only partially diminished by choline replacement. Choline restriction in the setting of 10% kcal protein also caused moderate ketosis and hepatic fat accumulation, which were again attenuated when choline was replete. Key effects of the 5% kcal protein diet – weight loss, hepatic fat accumulation, and mitochondrial ultrastructural disarray and bioenergetic dysfunction – were mitigated by choline repletion. These studies indicate that synergistic effects of protein restriction and choline deficiency influence integrated metabolism and hepatic pathology in mice when nutritional fat content is very high, and support the consideration of dietary choline content in ketogenic diet studies in rodents to limit hepatic mitochondrial dysfunction and fat accumulation.

## Introduction

Ketogenic diets confer a multitude of beneficial effects on health in experimental and clinical settings, including weight loss in obesity and the amelioration of metabolic syndrome [1–5], anticonvulsant activity [6,7], autism [8], neurodegenerative diseases including Parkinson’s and Alzheimer’s [9–11], cardiomyopathy [12], and they show promise as potential adjunctive therapy for cancers [13,14]. However, the mechanisms through which ketogenic diets transduce their pleiotropic effects, and the long-term physiological and metabolic alterations that may occur during adherence to ketogenic diets, are incompletely understood. In particular, it is not clear whether beneficial effects of ketogenic diets are related to the generation and/or metabolism of ketone bodies, or whether the low carbohydrate content itself supports a salutary metabolic and/or endocrine milieu [7]. Rodents have been used extensively to determine the physiological and metabolic responses to ketogenic diets [15–21]. However, because diet-induced ketonemia in rodents requires marked diminution of both carbohydrates and protein content, truly ketogenic diets for rodents consist of a macronutrient balance that humans do not ingest [22]. A widely used [8,12,15,16,18–21,23–25] micronutrient-supplemented ketogenic diet for rodents, Bio-Serv F3666, is very high in fat (93.3% kcal), very low in carbohydrate (1.8%), and relatively depleted of protein (4.7%). This diet provokes ketonemia, weight loss, and induces a hepatic gene expression signature consistent with decreased de novo lipogenesis and increased fatty acid oxidation [15,16]. C57BL/6J mice maintained on this ketogenic diet become lean, euglycemic, ketotic, hypoinsulinemic, and glucose intolerant [15,21]. Additionally, mice fed this ketogenic diet exhibit a distinctive nonalcoholic fatty liver disease (NAFLD) profile including micro- and macrovesicular steatosis with hepatocellular injury and repair. The macronutrient composition that induces this histological signature is atypical for human NAFLD, which is commonly associated with increased carbohydrate intake and activated de novo lipogenesis [16,26,27]. In fact, low carbohydrate diets in humans may improve NAFLD [26–28].

To establish safe and effective therapeutic nutritional approaches for diseases that may be responsive to low-carbohydrate diets, it will ultimately be important to understand the driver mechanisms responsible for favorable responses, and whether these responses and their underlying mechanisms can be nutritionally dissociated from concomitantly-triggered pathophysiological responses. A potential contributor to the liver fat accumulation and injury that are provoked by Bio-Serv F3666 [16,21], despite its salutary effects in other organ systems [8,12,20], is protein restriction. In fact, Bio-Serv F3666 diet may mimic a subset of the systemic and hepatic sequelae of malnutrition attributable to very low protein ingestion in human patients with kwashiorkor [29]. An additional prospective explanation is the influence of choline restriction [30]. While fully replete rodent diets are supplemented to contain ≥ 2 g/kg choline, Bio-Serv F3666 is not supplemented, and therefore contains only ~300 mg/kg from naturally-derived fat sources. In this study, we examined the roles of dietary protein and choline contents on the systemic and hepatic derangements that occur in the setting of very high fat, very low carbohydrate diets. Using Bio-Serv F3666 as a reference diet, we designed a very low carbohydrate, very low protein, and choline restricted (VLP/C^-^) diet and three additional diets which differ only in total protein [10% kcal (low protein, LP) vs. 5% kcal (very low protein, VLP)] and choline content [replete (C^+^) vs. restricted (C^-^)] to elucidate the contributing mechanistic roles of each component in the onset and progression of the hepatic pathology observed in ketogenic diet-fed mice.

## Methods

### Animals and diets

C57BL/6J wild-type mice were maintained on Lab Diet (5053) *ad libitum* and autoclaved water on cedar chip bedding at 22°C. Lights were off between 1800 and 0600. Beginning at the age of 6 weeks male mice were maintained for 6 weeks on one of the following very high fat, very low carbohydrate (ketogenic) paste diets, whose contents are summarized in Table 1: 1) low protein, choline replete (LP/C^+^, Harlan-Teklad TD.110291), 2) low protein, choline restricted (LP/C^-^, Harlan-Teklad TD.110406), 3) very low protein, choline replete (VLP/C^+^, Harlan-Teklad TD.110634), or 4) very low protein, choline restricted (VLP/C^-^, Harlan-Teklad TD.110633). Diets were sterilized prior to shipment, stored at 4°C, and administered every 2-3 days in autoclaved glass dishes on the cage bottoms. Consumption of diets was determined between weeks 2 and 4 of the administration period, and was performed by weighing paste diet mass prior to and after 48 h of feeding. Age-matched cohorts of mice were maintained on the standard low-fat, polysaccharide-rich chow pellets (Lab Diet 5053) to serve as controls. Consistent with the historically-used Bio-Serv F3666 diet, the contributing fat sources of all four ketogenic diets are lard and milk fat; casein is the protein source, and sucrose is the minimal carbohydrate source (~1% kcal). All diets were supplemented with vitamin mix AIN-76 and mineral mix AIN-76A. The distribution of fatty acids among all four diets is similar, with 46-52% originating from saturated, 38-41% from monounsaturated, and 7-17% from polyunsaturated fatty acids. Choline replete diets are supplemented with 5.0 g/kg supplied as choline bitartrate. All four diets are soy meal (phytoestrogen) free. All experiments were performed after protocol approval by the Animal Studies Committee at Washington University.

**Table 1:** Macronutrient composition of mouse diets used in this study.

Diet	Vendor (Catalog Number)	Carb % kcal	Fat % kcal	Protein % kcal	Choline g/kg (degree of restriction)	Methionine g/kg (degree of restriction)	Cholesterol mg/kg	kcal/g
Standard Chow	Lab Diet (5053)	62.1	13.2	24.6	2.0 (N/A)	7.0 (N/A)	141	3.4
LP/C^+^	Harlan Teklad (110291)	1.4	88.9	9.7	5.3 (0%)	7.4 (0%)	2500	7.3
LP/C^-^	Harlan Teklad (110406)	1.4	89.0	9.6	0.3 (88%)	4.4 (37%)	2500	7.2
VLP/C^+^	Harlan Teklad (110634)	1.3	94.1	4.6	5.3 (0%)	3.7 (47%)	2500	7.6
VLP/C^-^	Harlan Teklad (110633)	1.3	94.1	4.6	0.3 (>90%*)	2.2 (82%*)	2500	7.6

All diets are irradiated (to sterilize) before feeding. Values represent percentage of total kcal. Choline added to diets as choline chloride (Lab Diet) or choline bitartrate (Harlan Teklad). Methionine content is calculated based on the amino acid composition of casein, plus additional elemental methionine (for VLP/C^+^ and LP/C^+^). * Corrected for diminished mass of diet consumed.

### Metabolite and insulin measurements

Serum samples were acquired from animals that had been fasted for 4 h on fresh cedar chip bedding. Serum glucose, free fatty acids (FFA), β-hydroxybutyrate (βOHB), triacylglycerols (TAG), and insulin concentrations were measured as previously described [18]. Hepatic TAG, using a Folch extract of liver and biochemical quantification, were quantified as previously described [24]. Serum alanine aminotransferase (ALT) was measured using an assay from Teco Diagnostics, according to manufacturer’s instructions.

### Measurements of body composition

Percent body fat was determined in awake animals using an EchoMRI instrument (Echo Medical Systems, Houston, TX).

### Histology

For hematoxylin and eosin stains, livers (n=3 separate specimens from three different animals/diet condition) were collected from mice immediately post-sacrifice and fixed in 10% neutral buffered formalin. Tissue was embedded in paraffin, microtome-sectioned, stained, imaged; low power images were photographed using standard methods, while higher power images were acquired at 0.5 μm slice thickness using a Zeiss LSM 700 confocal microscope and Zeiss Zen software. For F4/80 immunostains (n=3 stained sections, derived from three separate animals/diet condition), rat anti-F4/80 (Abcam, Cambridge MA) was incubated for 1 h at room temperature on liver cryosections (diluted 1:100 in 5% bovine serum albumin (BSA) in phosphate buffered saline (PBS), followed by Alexa Fluor 594 conjugated goat anti-rat IgG (Invitrogen, 1:300 in 5% BSA/PBS), and counterstained with 4',6-diamidino-2-phenylindole (DAPI, 40 ng/mL in PBS for 5 min) as previously described [16]. All slides were examined and images were acquired at 0.5 μm slice thickness using a Zeiss LSM 700 confocal microscope and Zeiss Zen software. Using ImageJ, F4/80^+^ cells were quantified and normalized per 100 nuclei in each field; on average 350 DAPI positive nuclei were quantified in each 20X field.

### Transmission electron microscopy (TEM)

Livers (n=2 separate specimens from two different animals/diet condition) were fixed in a modified Karnovsky’s fixative of 3% glutaraldehyde and 1% paraformaldehyde in 0.1M sodium cacodylate buffer for at least 24 h, followed by a post-fix in 2% osmium tetroxide in 0.1 M sodium cacodylate buffer for 1 h prior to en bloc staining with 2% aqueous uranyl acetate for 30 min, dehydration in graded ethanols and embedding in PolyBed 812 (Polysciences, Hatfield,PA). Tissue blocks were sectioned at 90 nm thickness, then stained with Venable’s lead citrate and viewed with a JEOL model 1200EX electron microscope. Digital images were acquired using the AMT Advantage HR (Advanced Microscopy Techniques, Danvers, MA) high definition charge-coupled device, 1.3 megapixel TEM camera.

### Measurements of hepatic very low density lipoprotein (VLDL) secretion in vivo

Mice were fasted overnight on fresh cedar chip bedding with free access to drinking water. The following morning, mice were injected with 500 mg/kg Triton WR-1339 (tyloxapol, Sigma) [31], and blood was collected at 0, 2, 4 and 6 h. To measure hepatic VLDL secretion, serum TAG levels were measured at each time point as described above.

*Mitochondrial genome copy number*. gDNA was isolated from liver of mice on all diets using a DNeasy Blood & Tissue Kit (Qiagen). Genome copy number was quantified using real-time quantitative PCR using the ΔΔCt approach as described [18], normalized using primers against nuclear *Rpl32* (F: 5’- CCTCTGGTGAAGCCCAAGATC; R: 5’-TCTGGGTTTCCGCCAGTTT) and mitochondrial genome primers against *mt-Atp6* (F: 5’- GCCATTCCACTATGAGCTG; R: 5’-GTTCCTTGTGGAAGGAAGTG); *mt-Cox1* (F: 5’-GACTCCTACCACCATCATTTC; R: 5’-AGGTGGGTAGACTGTTCAT); *mt-Cox3* (F: 5’-GGATTCTTCTGAGCGTTCTATC; R: 5’-GGGACTTCTAGAGGGTTAAGT); *mt-Cytb* (F: 5’-CCTTCATACCTCAAAGCAACGA; R: 5’-GATAAGTAGGTTGGCTACTAGGATTCAGT).

### Mitochondrial respiration

Whole livers were excised, washed, and suspended in mitochondrial isolation medium (MIM; 300 mM sucrose, 0.2 mM EDTA, 10 mM HEPES, pH 7.4) containing BSA (1 mg/mL). The samples were rapidly homogenized on ice by using a Glas-Col dounce homogenizer, and centrifuged at 600*g* for 10 min at 4°C. The resulting supernatant, which contained mitochondria, was spun at 8,000*g* for 15 min at 4°C, the supernatant discarded, the mitochondrial pellet resuspended in 10 mL of ice-cold MIM-BSA, and the sample centrifuged again at 8,000*g* for 15 min at 4°C. The pellet was briefly washed in ice-cold MIM and resuspended in 75 μL of ice-cold MIM (pH 7.2) per 100 mg of liver. Total protein was quantified by Bradford assay (Bio-Rad). Respiration was quantified at 37°C using a water-jacketed Clark Electrode (Hansatech Instruments) under conditions described previously [32]. Briefly, 0.5 mg of mitochondria were added to 1 mL of respiration buffer [125 mM KCl, 20 mM HEPES, 3 mM Mg-acetate, 0.4 mM EGTA, 0.3 mM dithiothreitol (DTT), 5 mM KH_2_PO_4_, 0.2% BSA, pH 7.1] containing one of four substrate combinations: (i) 20 μM palmitoyl-L-carnitine and 5 mM malate, (ii) 5 mM succinate and 10 μM rotenone, (iii) 100 μM duroquinol and 10 μM rotenone, or (iv) 0.5 mM N,N,N′,N′-Tetramethyl-*p*-phenylenediamine (TMPD), 2 mM ascorbate, and 10 μM rotenone. Duroquinol was prepared using a method previously described [33]. Briefly, duroquinone was reduced using KBH_4_ under acidic pH and anoxic atmosphere. The solubility of oxygen in the respiration buffer at 37°C was 235 nmol O_2_ per mL. Following measurement of basal (state 2) respiration, ADP was added to the respiration chamber at a concentration of 1 mM in respiration buffer, and maximal (state 3) respiration quantified. Thereafter, state 4 (F_1_F_0_ ATPase-independent) respiration was measured by adding 1 μg/mL oligomycin (Sigma) to inhibit ATP synthase. Mitochondrial uncoupling was measured following the addition of 5 μM carbonyl cyanide 4-(trifluoromethoxy) phenylhydrazone (FCCP) where applicable.

### Statistical analyses

Analyses were performed with GraphPad software (Prism, San Diego, CA), using tests described within the text and figure legends.

## Results

### Comparison of choline replete and choline restricted, low- and very low protein diets on body composition and baseline metabolic parameters

To determine the effects of choline and protein content on systemic and hepatic metabolism in the setting of very high fat (≥89% kcal), very low carbohydrate (~1% kcal) macronutrient composition, cohorts of 6 week-old C57BL/6J mice, all with mean starting weights between 20.0 and 21.0 g, were fed one of four custom paste diets for 6 weeks, defined as (1) low protein (~10% kcal), choline replete (LP/C^+^), (2) low protein, choline restricted (LP/C^-^), (3) very low protein (~5% kcal), choline replete (VLP/C^+^), and (4) very low protein, choline restricted (VLP/C^-^), and we compared responses of these groups of mice to a cohort maintained on standard chow (Table 1). The VLP/C^-^ diet was formulated to replicate Bio-Serv F3666, a diet that we and others previously studied because it induces ketosis and weight loss in murine models [8,12,15,16,18–21,23–25]. Bio-Serv F3666 also causes hepatic steatosis, hepatocellular injury, and hepatic macrophage accumulation in C57BL/6J wild-type mice [16].

**Figure 1:**
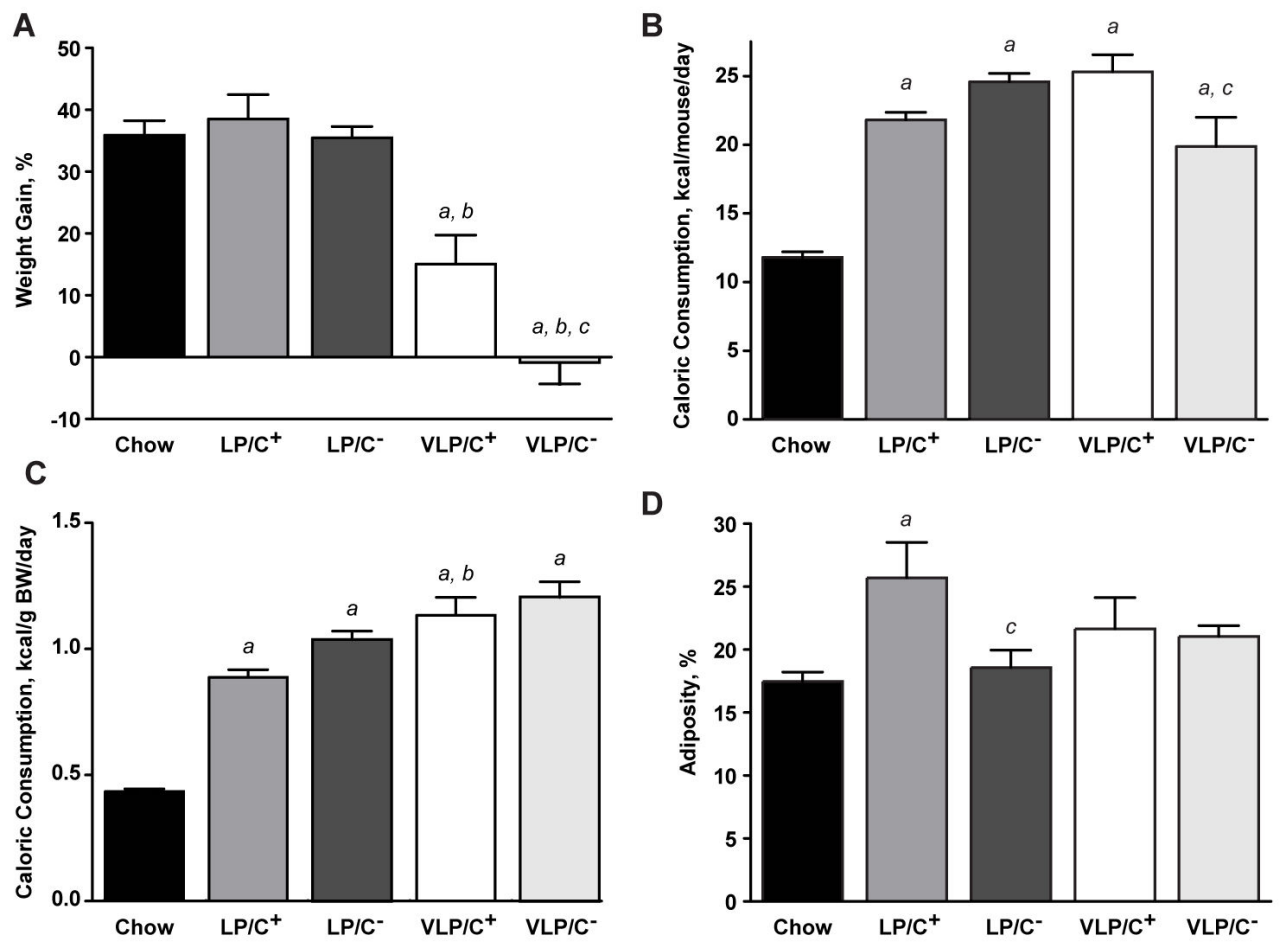
Metabolic parameters of mice maintained on very high fat, low protein, very low carbohydrate diets. (**A**) Body weight responses to 6 weeks of maintenance on the experimental paste diets, compared to chow controls. n=10-15 mice/group. a, p≤0.001; *b*, p≤0.001; *c*, p≤0.01. See end of this legend for description of the individual comparisons depicted by each letter. (**B**) Caloric consumption of diet, normalized per mouse, between weeks two and four of the 6 weeks of maintenance on the diets. n=5-10 mice/group. a, p≤0.001; *c*, p≤0.05. (**C**) Caloric consumption of diet, normalized per gram of body weight (BW). n=5-10 mice/group. a, p≤0.001; b, p≤0.01. (**D**) Percent adiposity after 6 weeks on each of the diets. n=5-10 mice/group. a, p≤0.01; *c*, p≤0.05. For all panels, data are presented as means±SEM. *a*, significantly different compared to chow; *b*, significant difference attributable to decrease in protein content (from 10% kcal to 5% kcal) at a fixed choline content; *c*, significant difference attributable to restriction in choline content (from 5.3 g/kg to 0.3 g/kg) at a fixed protein content; by 1-way ANOVA with Tukey’s post hoc testing.

As previously observed in mice maintained on Bio-Serv F3666, but unlike mice maintained on standard chow or the other three experimental diets, mice maintained on VLP/C^-^ diet for 6 weeks failed to gain weight (Figure 1A). While mice maintained on VLP/C^+^ gained weight over the 6 week period, the extent of weight gain was ~60% less than that of mice maintained on standard chow, LP/C^+^, or LP/C^-^. Differences in weight gain between VLP/C^+^ and VLP/C^-^ fed cohorts may be partially attributable to diminished caloric consumption by mice fed VLP/C^-^ (Figure 1B), but normalizing caloric consumption to body weight revealed no differences between these two diet cohorts (Figure 1C). Independent of whether caloric consumption was normalized per animal or to body mass, caloric intake was increased in all cohorts of mice fed these paste diets, compared to chow-fed mice. Together, these observations are consistent with increased energy expenditure of mice maintained on these very high fat diets (compared to standard chow), which was observed in studies of mice maintained on Bio-Serv F3666 [15,21]. Irrespective of choline content, LP diet-ingesting cohorts both gained significantly more weight than mice in the VLP-ingesting cohorts, indicating that increased protein content of these two diets supports weight gain [34]. Percent adiposity, measured by MRI, was commensurate with the chow-fed cohort for mice maintained on LP/C^-^, VLP/C^+^, and VLP/C^-^ (Figure 1D), while mice fed LP/C^+^ displayed a significant increase in adiposity relative to the other cohorts.

Compared to the chow-fed cohort, whose serum βOHB concentrations were 0.10±0.03 mM, all groups of mice fed the experimental diets for 6 weeks developed statistically significant ketosis (Table 2). Mice maintained on VLP/C^-^ exhibited the highest βOHB mean serum concentration, 1.46±0.34 mM (n=10). Increasing protein content had a modestly stronger suppressive effect on ketosis than replenishing choline: mice maintained on LP/C^-^ exhibited serum βOHB concentrations that were 2.4-fold lower compared to VLP/C^-^-fed mice (0.60+0.08 mM in LP/C^-^ mice; p=0.03 compared to the VLP/C^-^ group, n=10/group), while the addition of choline to VLP/C^-^ (i.e., the VLP/C^+^ diet) decreased ketosis only to 0.80±0.11 mM, which was not statistically different from that induced by VLP/C^-^. The ketosis-suppressing effect of increasing protein from 5% kcal to 10% kcal was also observed when comparing the two choline-replete paste diets: while serum βOHB concentrations were 0.80±0.11 mM in mice maintained on VLP/C^+^, this decreased to 0.44±0.03 mM in mice maintained on LP/C^+^ (p=0.006, n=10/group). Taken together, the greatest degree of ketosis was observed in mice maintained on VLP/C^-^, and the addition of choline to this diet did not abrogate ketosis, as mice maintained on VLP/C^+^ display an 8-fold elevation in serum βOHB concentrations compared to chow-fed mice (p<0.0001, n=8-11/group).

**Table 2:** Serum parameters after 6 weeks on diets.

Diet	βOHB, mM	Glucose, mg/dL	Insulin, ng/mL	HOMA-IR	FFA, mM	TAG, mg/dL
Standard Chow	0.10±0.03	121.6±3.7	1.10±0.14	8.2±1.1	1.24±0.10	70.3±4.5
LP/C^+^	0.44±0.03^*a*^	212.2±15.2^*aa*^	0.90±0.16	11.7±2.4	0.36±0.08^*aaa*^	67.3±3.1
LP/C^-^	0.60±0.08^*a*^	175.8±11.6^*a*^	0.70±0.10	8.6±1.8	0.30±0.06 ^*aaa*^	57.3±3.8
VLP/C^+^	0.80±0.11^*a*,*b*^	172.5±8.9^*a*^	0.62±0.12	7.1±1.5	0.48±0.06 ^*aaa*^	85.7±8.5
VLP/C^-^	1.46±0.34^*aaa,bb*^	119.0±15.8^*b*,*c*^	0.37±0.04 ^*aa,b*^	2.9±0.5^*a*,*b*^	0.64±0.05 ^*aaa,b*^	95.0±10.7 ^*bb*^

HOMA-IR, homeostatic model assessment of insulin resistance

FFA, non-esterified(free) fatty acids

TAG, triacylglycerols

^*a*^ p≤0.05, ^*aa*^ **p≤0.01, ^*aaa*^ p≤0.001 (one-way ANOVA) compared to chow

^*b*^ p≤0.05, ^*bb*^ p≤0.01 (one-way ANOVA) attributable to decrease in protein content (from 10% kcal to 5% kcal) at a fixed choline content

^*c*^ p≤0.05 (one-way ANOVA) attributable to restriction in choline content (from 5.3 g/kg to 0.3 g/kg) at 5% kcal protein

Consistent with our previous findings in Bio-Serv F3666-fed mice, VLP/C^-^-fed mice exhibited 4 h-fasted serum glucose levels commensurate with chow-fed animals, despite exhibiting 2.9-fold lower serum insulin concentrations (p<0.0001, n=5-10/group) (Table 2) [16]. Conversely, LP/C^+^-, LP/C^-^-, and VLP/C^+^-fed mice each exhibited significantly elevated serum glucose levels relative to both chow-fed and VLP/C^-^-fed cohorts. Only VLP/C^-^-fed mice exhibited markedly diminished serum insulin concentrations compared to those of chow-fed mice, which accounts for the markedly decreased HOMA-IR index in this cohort (2.9±0.5 vs. 8.2±1.1 in VLP/C^-^ and chow-fed mice, respectively, p=0.0003, n=5-10/group). Additionally HOMA-IR indices of VLP/C^-^-fed mice are significantly lower than those of mice fed LP/C^-^ (8.6±1.8, p=0.007, n=10/group). Circulating non-esterified (free) fatty acids (FFA) were diminished in mice fed each of the paste diets compared to chow-fed controls, and these concentrations were inversely proportional to dietary protein (Table 2). Likewise, circulating TAG concentrations were inversely proportional to protein content, with less of an effect derived from choline.

### Effects of dietary choline and protein content on hepatic steatosis and histopathology

**Figure 2:**
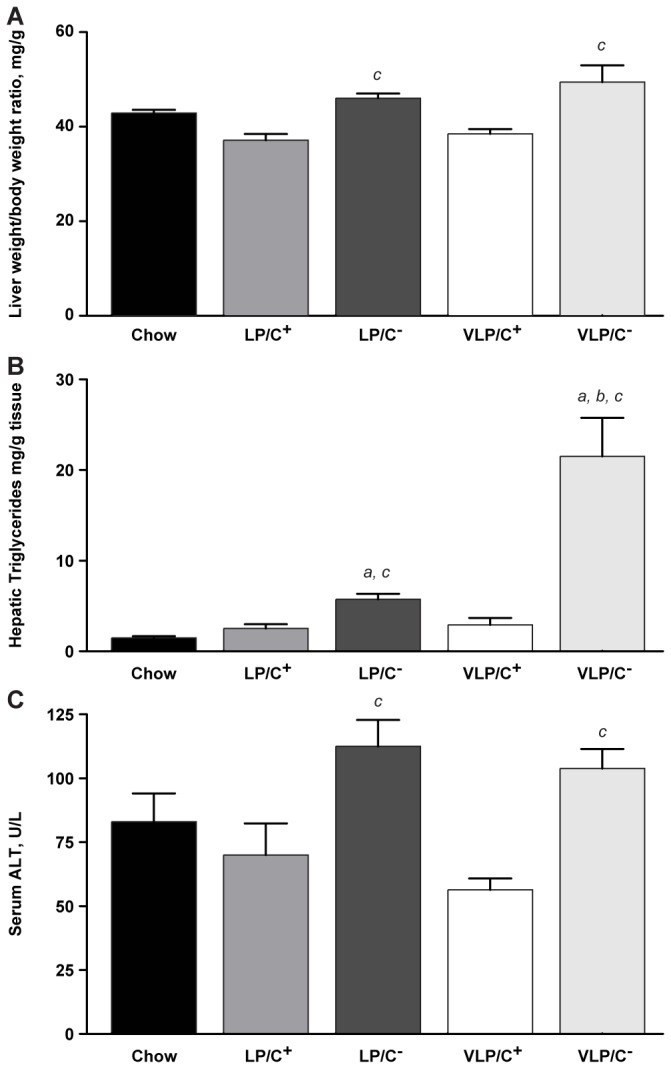
Roles of varying protein and choline nutrient contents in very high fat, low protein, very low carbohydrate diet-induced hepatic steatosis. (**A**) Liver weight/body weight ratios after 6 weeks on the diets. *n*=5-10 mice/group. c, p≤0.05 for LP/C^-^ and p≤0.01 for VLP/C^-^. See end of this legend for description of the individual comparisons depicted by each letter. (**B**) Biochemical quantification of hepatic TAG content, normalized to liver mass. *n*=5-10 mice/group. a, p≤0.05 for LP/C^-^ and p≤0.01 for VLP/C^-^; *b*, p≤0.05; *c*, p≤0.05 for LP/C^-^ and p≤0.01 for VLP/C^-^. (**C**) Serum alanine aminotransferase (ALT) concentrations. n=4-6 mice/group. c, p≤0.05. Data are presented as means±SEM. *a*, significant difference compared to chow; *b*, significant difference attributable to decrease in protein content (from 10% kcal to 5% kcal) at a fixed choline content; *c*, significant difference attributable to restriction in choline content (from 5.3 g/kg to 0.3 g/kg) at a fixed protein content; by 1-way ANOVA with Tukey’s post hoc testing.

To determine the effects of dietary protein and choline levels on hepatic structure and function, we quantified hepatic mass and intrahepatic triglyceride content after 6 weeks on each diet. Mice fed either choline restricted diet (VLP/C^-^ or LP/C^-^) exhibited liver weight to body weight ratios that were ~25% increased compared to mice fed the choline replete diets (LP/C^+^ and VLP/C^+^) (Figure 2A). Additionally, normalized liver weights of mice fed either choline replete paste diet were ~13% smaller compared to chow-fed mice. Hepatic TAG content in mice maintained on VLP/C^-^ (21.5±4.2 mg/g tissue) was markedly higher than that of mice from any of the other paste diet cohorts (Figure 2B). Hepatic TAG content in mice maintained on the LP/C^-^ diet was significantly higher than those of mice maintained on LP/C^+^, VLP/C^+^, or chow control, indicating that choline restriction in the context of a very high fat diet predisposes to increased hepatic TAG content and that this is exacerbated by severe protein limitation. Serum alanine ALT activities were significantly elevated in both cohorts maintained on the choline restricted diets, independent of protein content (Figure 2C).

**Figure 3:**
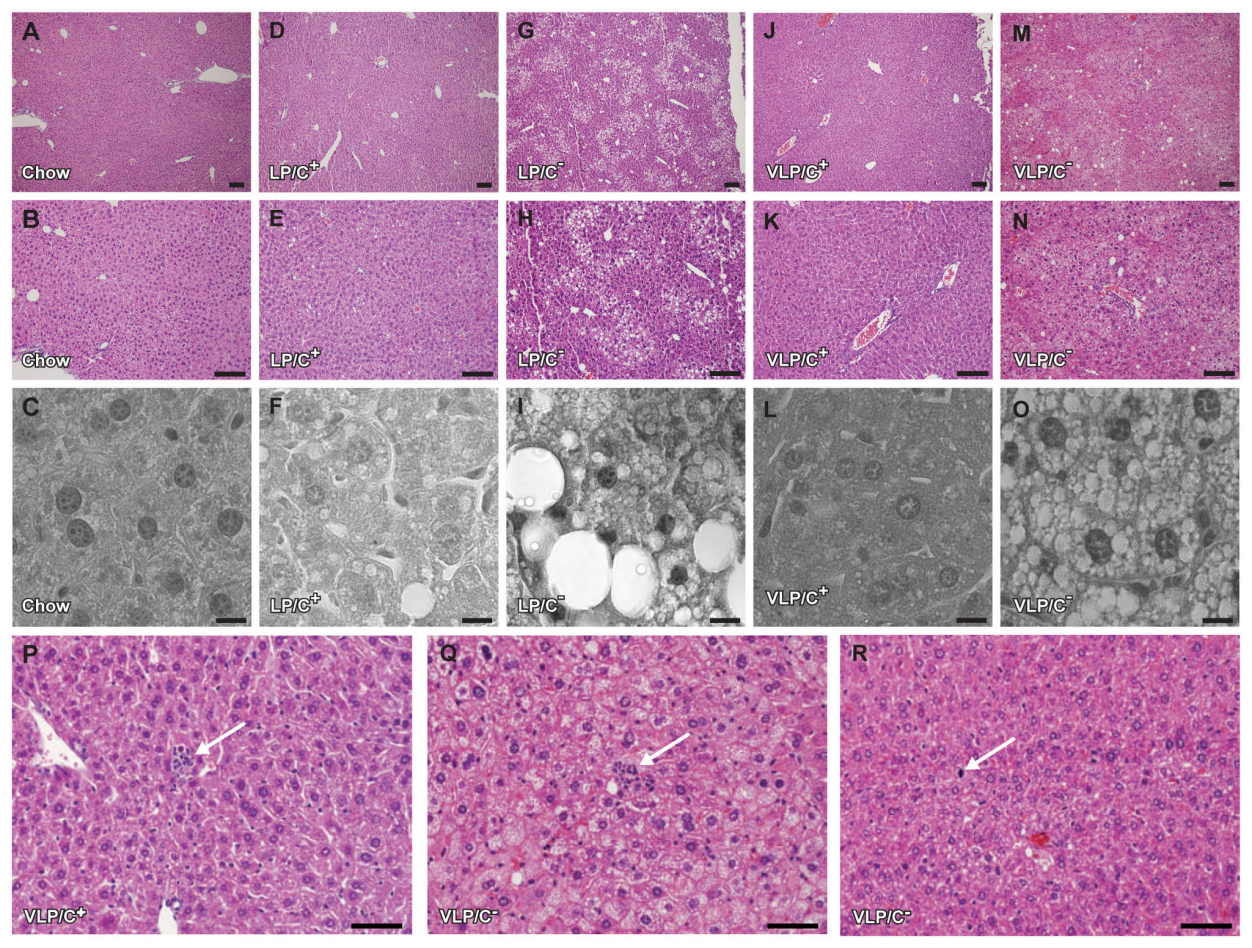
Intrahepatic triglyceride content and hepatic histopathology in mice fed very high fat, low protein, very low carbohydrate diets. Hepatic histology in mice maintained for 6 weeks on (**A**–**C**) standard chow; (**D**–**F**) LP/C^+^ diet, which caused very small amounts of mixed large and small droplet steatosis in hepatocytes restricted to zone 2 (a representative example of zone 2 is displayed in panel **F**), and no inflammation; (**G**–**I**) LP/C^-^ diet, which caused mixed large and small droplet macrovesicular steatosis in a zone 2 distribution (a representative example of zone 2 is displayed in panel **I**); (**J**–**L**) VLP/C^+^ diet, which exhibited small lipid droplets only at higher power; (**M**–**O**) VLP/C^-^ diet, which caused diffuse steatosis that is predominantly small and microvesicular with some macrovesicular droplets. Numerous clusters of inflammatory cells, some of which are likely associated with necrotic hepatocytes, were observed. Livers of mice fed both VLP/C^+^ (**P**) and VLP/C^-^ (**Q**) exhibit inflammatory foci (arrows). (**R**) Only in livers from VLP/C^-^-fed were mitotic figures observed (arrow). Scale bars: (**A, B, D, E, G, H, J, K, M, N**, lower power images taken with standard light microscopy, original magnification at 10X or 20X), 100 μm; (**C, F, I, L, O**, higher power images taken with confocal microscopy, original magnification at 80X), 10 μm; (**P, Q, R**, medium power images taken with standard light microscopy, original magnification at 40X), 50 μm.

To determine the histopathological changes that accompany hepatic lipid accumulation in mice maintained on these ketogenic diets, liver sections from cohorts maintained on each of the diets were stained with hematoxylin and eosin. Liver sections from chow-fed animals displayed normal architecture and were devoid of steatosis and inflammatory cell infiltration (Figure 3A–C). Livers from mice maintained on LP/C^+^ exhibited very small amounts of mixed large and small droplet steatosis in hepatocytes located exclusively in acinar zone 2; periportal and perivenular hepatocytes were virtually free of steatosis (Figure 3D–F). Mice fed the choline-restricted counterpart, LP/C^-^, displayed a marked increase in mixed large and small droplet macrovesicular steatosis in a zone 2 distribution, between the portal tracts and terminal hepatic venules (Figure 3G–I).

**Figure 4:**
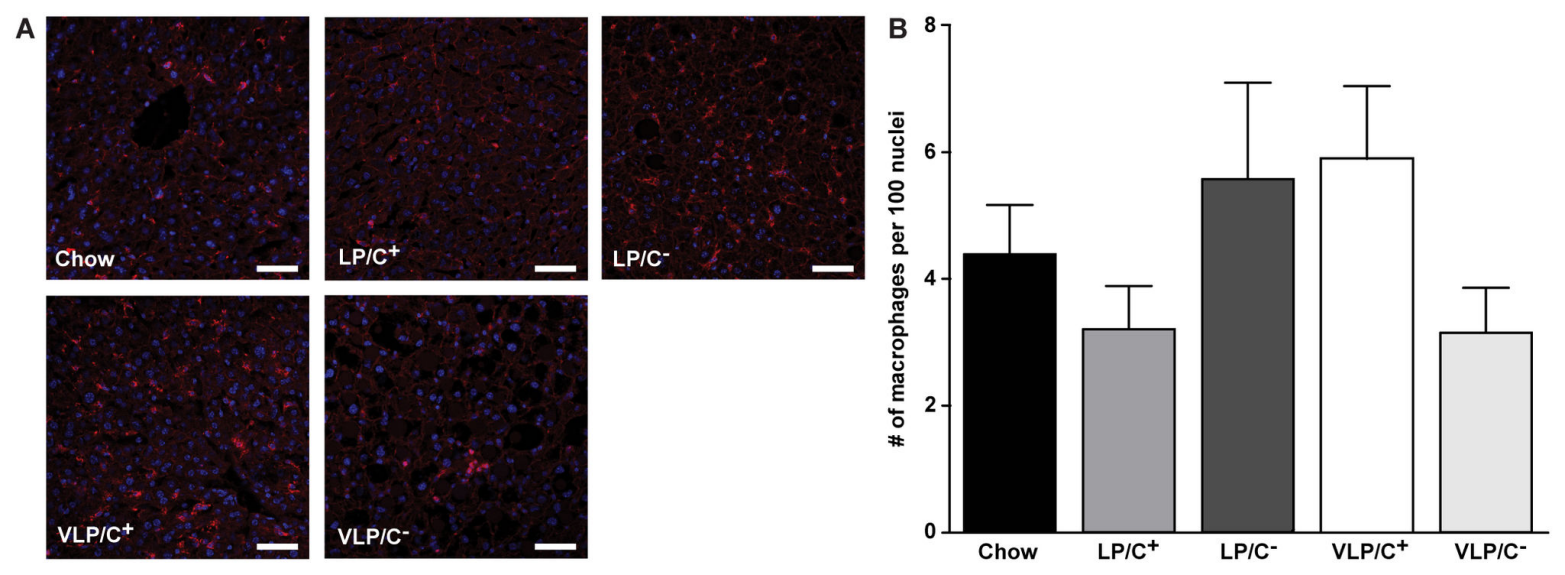
Hepatic macrophage density in mice fed very high fat, low protein, very low carbohydrate diets. (**A**) Confocal images of F4/80^+^ macrophages (scale bars, 50 μm) and (**B**) quantification of F4/80^+^ macrophages normalized to the number of DAPI-stained nuclei from liver sections of mice maintained on the indicated diets for 6 weeks. Data are presented as means±SEM. n=3 mice/group with n=3 20X fields quantified per section/mouse.

Low power images from livers from mice fed VLP/C^+^ were unremarkable, with no signs of steatosis (Figure 3J-K), while higher power images revealed scant small droplet steatosis in hepatocytes in acinar zone 2 (Figure 3L). Conversely, livers from mice maintained on VLP/C^-^ diet displayed diffuse and extensive steatosis (Figure 3M–O), primarily of small droplet macrovesicular and microvesicular morphologies. Diffuse activation of sinusoid-lining cells, characterized by increased numbers of intrasinusoidal nuclei, and occasional acidophil bodies were also noted, but no zonal necrosis was observed. While differing markedly in hepatic lipid content, inflammatory foci were readily observed in higher power images of hepatic sections from both VLP/C^+^- and VLP/C^-^-fed mice (Figure 3P-Q). Mitotic figures, an additional sign of hepatocellular injury, were observed uniquely in livers from VLP/C^-^-fed mice (Figure 3R). While immunostaining for F4/80^+^ macrophages revealed modest differences in macrophage density (per total number of cells observed), none of these differences was statistically significant (Figure 4). Moreover, abundances of the mRNAs encoding neither the macrophage marker CD68 nor the inflammatory maker TNFα differed among the diets (data not shown). While the total macrophage density trended lower in mice fed VLP/C^-^, F4/80^+^ cell clusters were observed in livers sections from VLP/C^-^-fed mice, but not in hepatic sections from mice fed the other diets (Figure 4A).

### Very low carbohydrate/very high fat diets impair VLDL secretion

**Figure 5:**
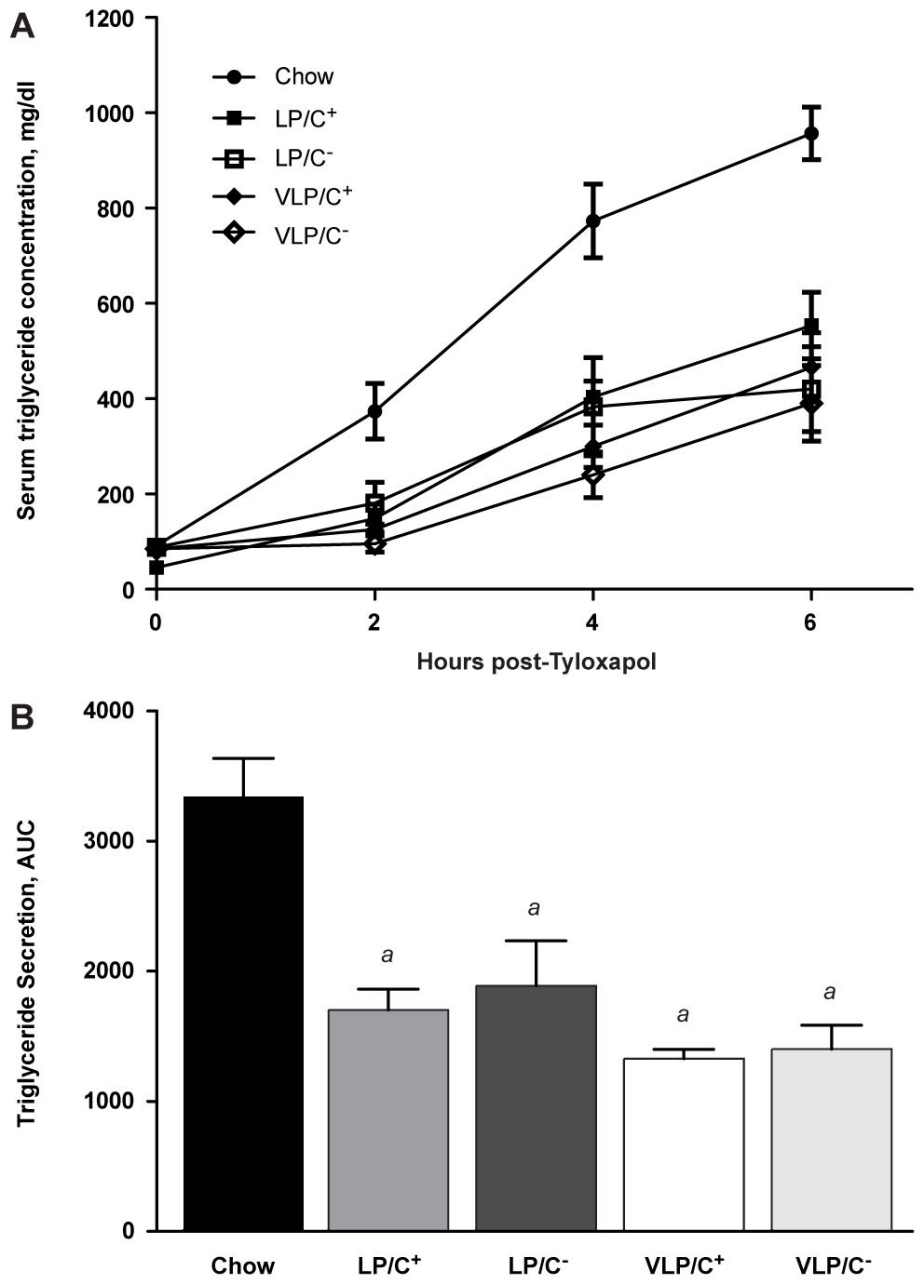
Hepatic TAG secretion in mice fed very high fat, low protein, very low carbohydrate diets. Mice from each dietary group (6 weeks each diet) were fasted for 18 h. Blood was collected prior to (0 h) and after intraperitoneal injection of tyloxapol. (**A**) Serum TAG concentration and (**B**) areas under the curve (AUC), *n*=5 mice/group. Data are presented as means±SEM. *a*, significantly different compared to chow, *p*≤0.001 by 1-way ANOVA with Tukey’s post hoc testing.

Choline deficiency has been linked to impaired VLDL packaging and secretion in the setting of high carbohydrate diets [31]. Because choline is an essential nutrient required for the formation of phosphatidylcholine (PC), and PC biosynthesis is required for the formation of VLDL particles for egress of TAG from the liver [35], we determined whether the increased hepatic TAG content of mice maintained on these choline restricted diets was attributable to impaired VLDL secretion by quantifying serum TAG content after overnight-fasted mice had been administered the lipoprotein lipase inhibitor tyloxapol. Surprisingly, while all groups demonstrate significantly impaired VLDL secretion relative to chow-fed control mice, no differences among the four paste diet-fed cohorts were observed (Figure 5).

### Influence of dietary choline on mitochondrial structure, number, and function in very low protein and carbohydrate, very high fat diets

**Figure 6:**
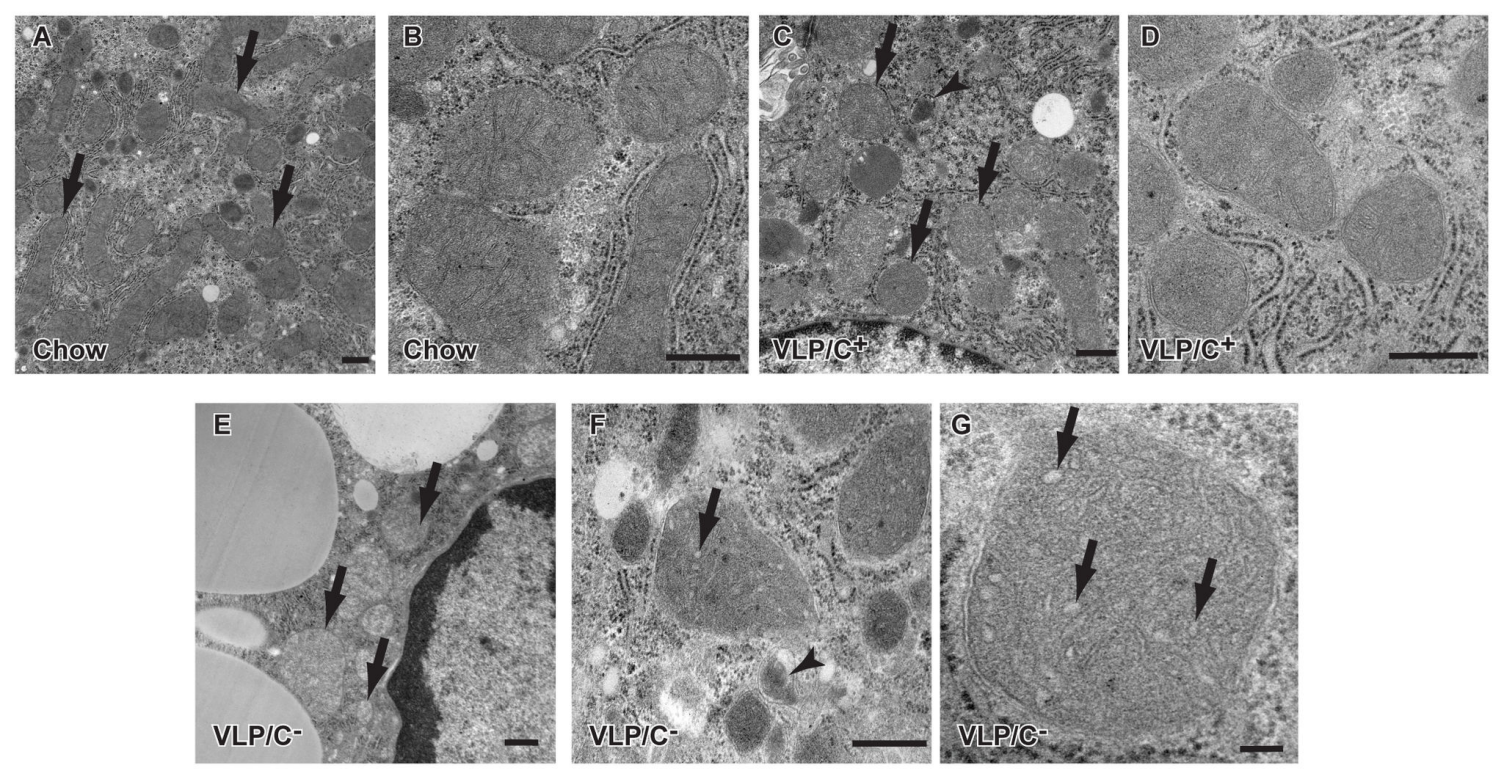
Abnormal mitochondrial ultrastructure in mice fed a choline restricted, very high fat, low protein, very low carbohydrate diet. (**A**) Transmission electron micrograph of hepatocytes from mice maintained for 6 weeks on standard chow reveals normal mitochondrial structure. Arrows, mitochondria. (**B**) Higher power image of mitochondria from mice maintained on standard chow, demonstrating normal cristae. (**C**) Hepatocyte mitochondria from livers of mice maintained on VLP/C^+^ exhibited normal cristae folding and evident double membranes. Sparse microvesicular lipid droplets were also evident (white circular structure). Arrows, mitochondria; arrowhead; autophagosome. (**D**) Higher power image of hepatocyte mitochondria from mice maintained on VLP/C^+^, showing morphology of the cristae. (**E**) VLP/C^-^ diet induces massive hepatocyte steatosis (note large circular pale fat droplets), and swollen mitochondria with disorganized and dilated cristae. Hepatocyte nucleus is on the right side of the image. Arrows, mitochondria. (**F**) Higher power image of hepatocyte mitochondria from mice maintained on VLP/C^-^, with dilated cristae (arrow) and an autophagosome (arrowhead). (**G**) High power image of hepatocyte mitochondria from mice maintained on VLP/C^-^, with dilated cristae (arrows). Scale bars, 500 nm (**A**–**F**), 100 nm (**G**).

Among the paste diet cohorts, mice maintained on the VLP/C^+^ and VLP/C^-^ diets exhibited the greatest difference in intrahepatic fat content, both histologically and biochemically, despite similar VLDL secretion capacity and equal caloric contents of protein, carbohydrate, and fat. Because adequate choline content is essential for normal mitochondrial structure and function in other macronutrient contexts [36–40], we determined whether choline restriction in the VLP dietary context was associated with abnormalities of mitochondrial ultrastructure. As expected, transmission electron microscopy of liver sections from chow-fed mice revealed that hepatocyte mitochondria exhibit smooth outer and inner membranes with tightly-organized cristae (Figure 6A-B). Most hepatocyte mitochondria of VLP/C^+^-fed mice also exhibited normal ultrastructure, although a small subset of hepatocyte mitochondria lacked organized cristae (Figure 6C-D). Moreover, sparse microvesicular lipid droplets were evident in hepatocytes of VLP/C^+^-fed mice, a feature that was not observed in hematoxylin and eosin-stained sections for light microscopy. Unlike hepatocyte mitochondria in chow-fed and VLP/C^+^-fed mice, all hepatocyte mitochondria of VLP/C^-^-fed mice exhibited swollen and disorganized cristae, and many lacked definitive cristae entirely (Figure 6E–G). Additionally, the spatial organization of mitochondria within the cytoplasm was markedly disrupted by the presence of extensive lipid droplets in hepatocytes of VLP/C^-^-fed mice.

**Figure 7:**
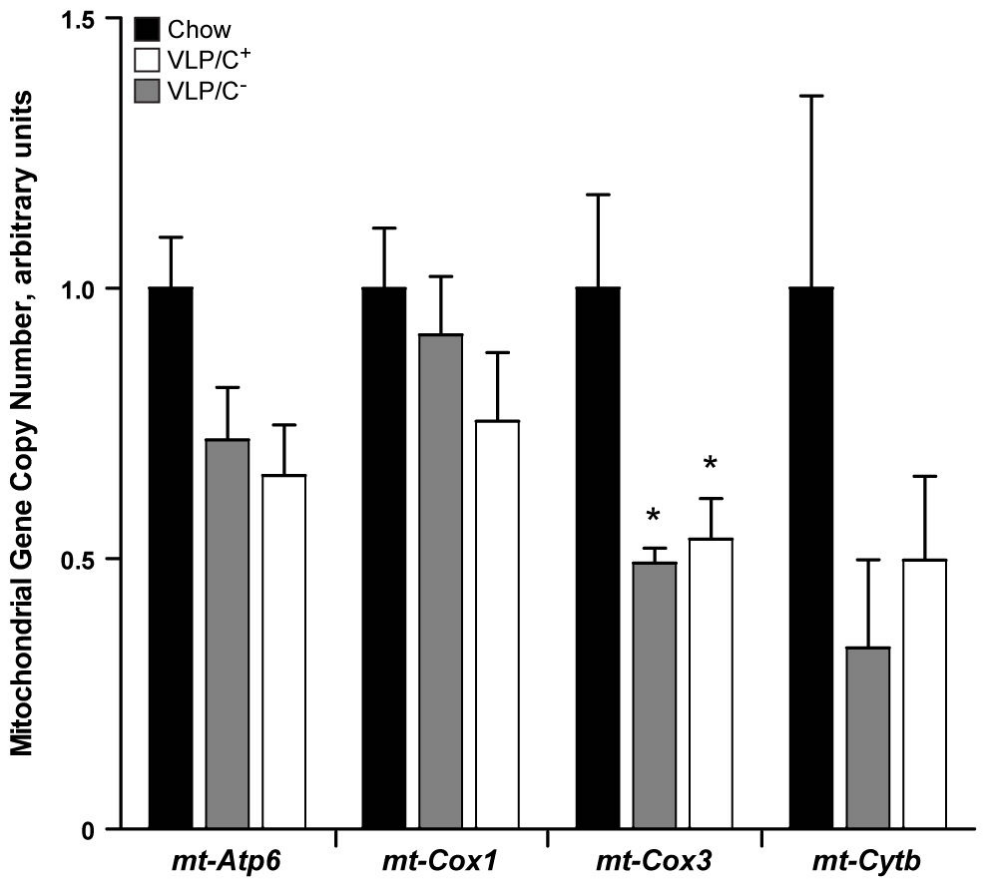
Relative hepatic mitochondrial genome content in mice fed very low protein and carbohydrate, very high fat diets. Quantification of mitochondrial genome copy number (relative abundance) by qPCR using purified liver gDNA from mice maintained on the diets for 6 weeks. Data are presented as means±SEM; n=4-5/group, *p≤0.05 by 1-way ANOVA with Tukey’s post hoc testing.

To further probe whether impairments in mitochondrial function contribute to the extensive lipid accumulation observed in mice fed the VLP/C^-^ diet, we quantified the effects of choline content in the VLP dietary contexts on hepatic mitochondrial DNA content. Relative abundance of the mitochondrial genome (mtDNA), normalized to the nuclear genome (nDNA) was quantified by qPCR using isolated liver genomic DNA from livers of chow-, VLP/C^+^, and VLP/C^-^-fed mice. Primers targeting *mt-Atp6, mt-Cox1, mt-Cox3*, and *mt-Cytb* were independently employed as reporters of mtDNA relative abundance, normalized against the nDNA gene *Rpl32* (Figure 7). While variation in the normalized mtDNA content was observed, depending on the mtDNA gene quantified, livers of mice fed both VLP/C^+^ and VLP/C^-^ exhibited statistically significant diminutions in mtDNA content, relative to chow-fed control when using *mt-Cox3* as a marker, with similar trends observed using the other three mtDNA markers.

To determine if the marked diminution in hepatic lipid content in VLP/C^+^-fed mice could be linked to alterations in mitochondrial bioenergetics, mitochondria were isolated from livers of mice maintained for 6 weeks on chow, VLP/C^+^, or VLP/C^-^ diets, and respiration studies were performed. Substrates palmitoyl-L-carnitine + malate (which donate electrons to Complexes I and II of the electron transport chain), succinate + rotenone (rotenone is a Complex I inhibitor, allowing electrons from succinate to be delivered exclusively to Complex II), duroquinol (which selectively donates an electron to Complex III), and TMPD (which selectively donates an electron to Complex IV) + ascorbate (an antioxidant that preserves TMPD’s reduced state) were independently used to probe bioenergetic function.

**Figure 8:**
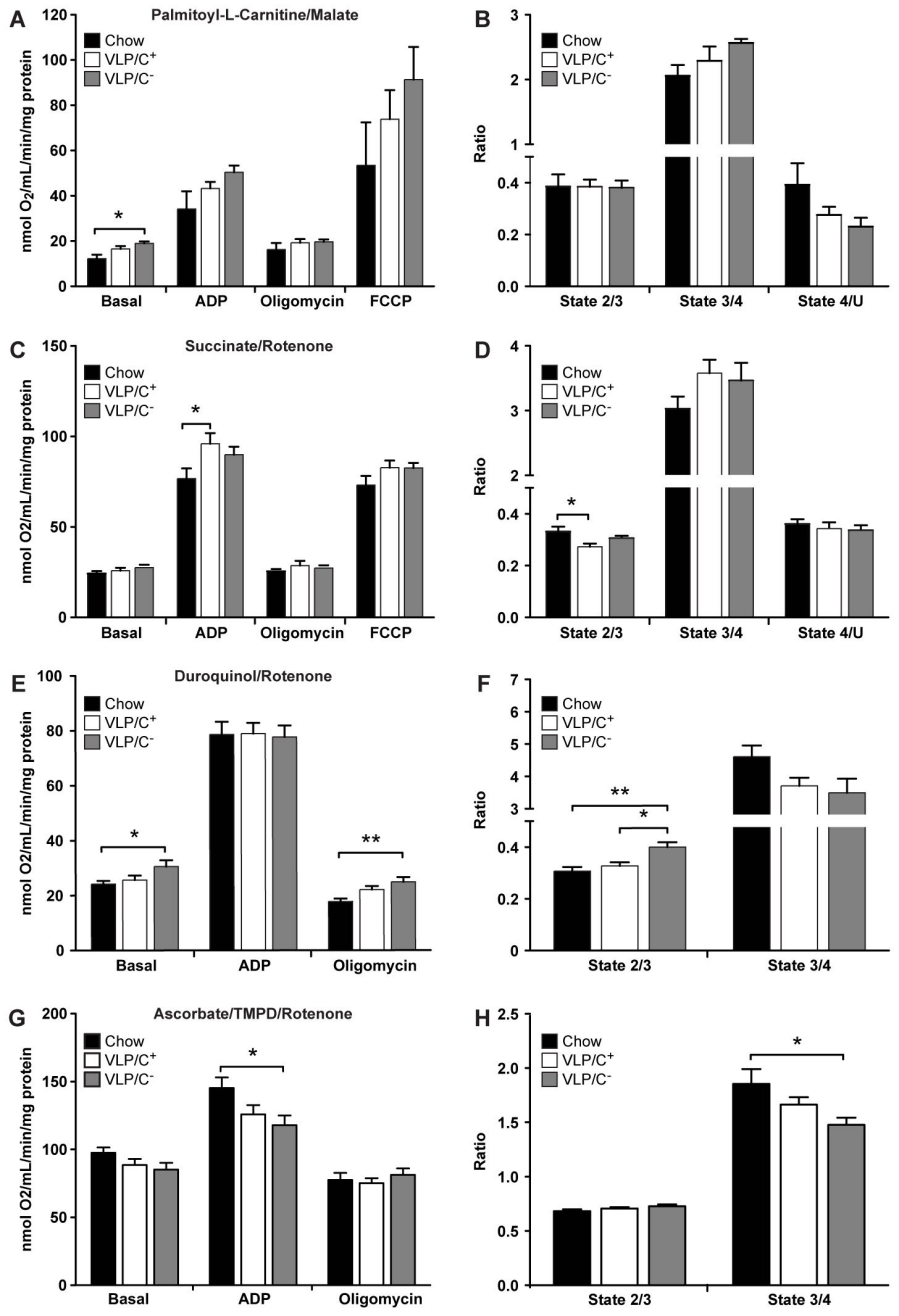
Respiration studies of hepatic mitochondria isolated from mice fed very low protein and carbohydrate, very high fat diets. (**A**) Respiration rates in the basal leak condition (state 2), ADP-stimulated condition (state 3), F_1_F_0_-ATPase independent condition (state 4), and uncoupled condition in hepatic mitochondria isolated from chow-fed, VLP/C^+^-fed, and VLP/C^-^-fed (for 6 weeks) mice using palmitoyl-L-carnitine and malate as substrates. *n*=4 mice/group. (**B**) Relative respiratory ratios of basal leak (state 2/state 3), respiratory control (RCR, state 3/state 4), and coupling control (CCR, state 4/uncoupled), derived from panel A. (**C**) Respiration rates in states 2-4 and while uncoupled in hepatic mitochondria isolated from chow-, VLP/C^+^, and VLP/C^-^-fed mice that respired using the Complex II-electron donor substrate succinate in the presence of rotenone (Complex I activity inhibitor). n=9 mice/group. (**D**) Relative respiratory ratios of state 2/state 3, state 3/state 4, and state 4/uncoupled, derived from panel C. (**E**) Respiration rates in states 2-4 in hepatic mitochondria isolated from chow-, VLP/C^+^, and VLP/C^-^-fed mice that respired using the Complex III-donor substrate duroquinol plus rotenone. n=9 mice/group. (**F**) Relative respiratory ratios of state 2/state 3, state 3/state 4, and state 4/uncoupled, derived from panel E. (**G**) Respiration rates in states 2-4 in hepatic mitochondria isolated from chow-, VLP/C^+^, and VLP/C^-^-fed mice that respired using the Complex IV-donor substrate combination TMPD/ascorbate, plus rotenone. n=9 mice/group. (**H**) Relative respiratory ratios of state 2/state 3, state 3/state 4, and state 4/uncoupled, derived from panel G. Data are presented as means±SEM. *p≤0.05; **p≤0.01 by 1-way ANOVA with Tukey’s post hoc testing.

Oxygen consumption in state 2 (basal proton leak) was significantly increased in hepatic mitochondria isolated from VPL/C^-^-fed mice that were tested using palmitoyl-L-carnitine + malate, compared to mitochondria prepared from livers of chow-fed mice (Figure 8A). However, while trends toward increased respiratory rates were observed, no significant differences among state 3 (ADP-stimulated), state 4 (F_1_F_0_ ATP-synthase inhibited), or uncoupled respiration (provoked by addition of the ionophore FCCP) were observed among the diets using palmitoyl-L-carnitine + malate as substrates (Figure 8A). Likewise there were no significant differences among the ratios of states 2/3 (basal leak/stimulated ratio), 3/4 (Respiratory Control Ratio, RCR), or state 4/uncoupled (Coupling Control Ratio, CCR) (Figure 8B). A trend toward decreased CCR was observed in the VLP/C^-^ group.

In the presence of succinate + rotenone, hepatic mitochondria from VLP/C^+^-fed mice exhibited significantly increased ADP-stimulated (state 3) oxygen consumption (Fig. 8C), which likely accounts for a significantly diminished relative basal leak ratio (Fig. 8D), compared to chow-fed mice. Duroquinol-stimulated respiration revealed that Complex III-associated basal leak and oligomycin-inhibited respiration were increased selectively in hepatic mitochondria isolated from VLP/C^-^-fed mice (Fig. 8E-F). Finally, respiratory rates in mitochondria stimulated with TMPD + ascorbate revealed that hepatic mitochondria from VLP/C^-^-fed mice exhibited significant impairment in ADP-stimulated oxygen consumption, compared to hepatic mitochondria from chow-fed control mice (Fig. 8G), and consequently, significantly diminished RCR compared to mitochondria from chow-fed animals (Fig. 8H). Using TMPD, mitochondria from VLP/C^-^-fed mice also exhibited a strong trend toward decreased RCR, when compared to hepatic mitochondria from VLP/C^+^-fed mice (p=0.06, n=9/group). Taken together, these results indicate that choline restriction in the setting of a high fat, very low carbohydrate diet contributes to abnormal hepatic mitochondrial function, potentially impairing terminal oxidation of fatty acids and contributing to hepatic TAG accumulation. It is important to note that the deficiencies of mitochondrial coupling and efficiency exhibited by isolated hepatocyte mitochondria from VLP/C^-^-fed mice likely underestimate in vivo bioenergetic dysfunction, because an enriched population of relatively intact mitochondria is assayed using readily accessible experimental substrates. Therefore integration of the mitochondrial structure and disordered spatial organization with these respiration studies suggests significantly impaired mitochondrial function in hepatocytes of VLP/C^-^-fed mice.

## Discussion

The driver mechanisms for beneficial effects of low carbohydrate ketogenic diets still remain to be elucidated, and the prospective roles of these diets in preventing or ameliorating human NAFLD are not defined. A commonly used rodent high fat, and very low protein, very low carbohydrate ketogenic diet, Bio-Serv F3666, has been employed to measure the effects of the ketogenic nutrient milieu on integrated metabolic homeostasis, and has also been used to mitigate abnormal phenotypes in the nervous and cardiovascular systems in mutant mouse strains. However, this diet also induces hepatic steatosis, inflammation and hepatocyte injury and repair in mice. To determine the underlying nutritional determinants of these adverse effects, we replicated this formulation by generating the VLP/C^-^ diet, which recapitulated the integrated metabolic and hepatic histopathological responses to Bio-Serv F3666 in C57BL/6J mice, and then tested three additional diets that systematically varied protein and choline content in VLP/C^-^. We observed that while protein and choline restriction synergistically contribute to the integrated metabolic phenotypes provoked by a high fat, very low carbohydrate diet in mice, choline restriction in this setting stimulated hepatic fat accumulation to a greater extent than protein restriction. Replenishment of choline in the VLP diet (i.e., comparing VLP/C^-^ to VLP/C^+^), markedly diminished hepatic fat accumulation, while increasing dietary protein content to 10% kcal in the setting of restricted choline (comparing VLP/C^-^ to LP/C^-^) exhibited a smaller mitigating effect on hepatic steatosis. Adding protein to a choline-replete diet (comparing VLP/C^+^ to LP/C^+^) exerted marginal additional effect on hepatic histopathology. While subtle variations were evident in histopathological inflammation and in serological evidence of hepatocellular injury (elevated ALT) among mice maintained on the four paste diets, significant differences in the hepatic density of F4/80^+^ macrophages after 6 weeks on the diets were not observed. We previously demonstrated that mice maintained on Bio-Serv F3666 (most similar to VLP/C^-^) exhibit an increased density of hepatic F4/80^+^ macrophages after 12 weeks on the diet [16]. Prolonged exposure to these high fat diets likely exacerbates hepatic inflammation through mechanisms summarized elsewhere [26]. Only VLP/C^-^, which caused the greatest degree of fat accumulation, induced histopathological evidence of hepatocyte regeneration at the 6 week time point, suggesting that this diet provokes the greatest degree of hepatocellular injury. Taken together, these findings suggest that inflammation and hepatocellular injury are likely secondary to the extent and duration of the metabolic abnormalities imposed by the nutrient contents.

Experiments in rodents have revealed molecular underpinnings of liver fat accumulation using methionine and choline deficient (MCD) diets [30,41], and diminished choline intake has also been associated with a more aggressive NAFLD course in humans [42]. The ability of experimental MCD diets to trigger steatosis and liver injury is facilitated by abundant mono- or disaccharides in the diet, because replacing these simple carbohydrates with starch markedly ameliorates the adverse histopathological effects of the MCD diet, a portion of which is hypothesized to occur through increased de novo lipogenesis [31,43]. Our results indicate that choline restriction also triggers hepatic steatosis and hepatocellular injury during a nutritional state in which the mediators of de novo lipogenesis are transcriptionally silenced due to very high fat content in the diet and diminished circulating insulin concentrations. The metabolism of choline has been mechanistically linked in mice to VLDL packaging and secretion through its direct contribution to PC synthesis [35]. Genetic models indicate that metabolic procession of choline through the phosphatidylethanolamine (PE) N-methyltransferase pathway, which synthesizes methionine from homocysteine and choline-derived methyl groups, is also an important contributor to VLDL secretion [35,44]. However, in our experiments, while hepatic VLDL secretion was impaired in mice maintained on all the paste diets, no additive effect of choline restriction was observed in these macronutrient contexts. It is therefore unlikely that an impairment of VLDL secretion explains the marked fatty liver phenotype of VLP/C^-^-fed mice.

Mitochondrial dysfunction is a known contributor to NAFLD pathogenesis [26,45]. Thus, we assessed hepatic mitochondrial structure and function in mice fed these very high fat diets. In mice maintained on VLP/C^-^ diet, we observed chaotic mitochondrial ultrastructure, including swollen cristae and many degenerating mitochondria. Choline repletion in the VLP diet was associated with improved mitochondrial ultrastructure and respiratory coupling and capacity in isolated mitochondria. However, choline repletion in the VLP diet did not correct the reduction in mitochondrial DNA content. Nonetheless, choline repletion may partially mitigate mitochondrial dysfunction by contributing to PC synthesis. PC is a major constituent of all cellular membranes, including mitochondria [36,37]. Though the exact phospholipid composition of membranes varies among experimental contexts, PC and PE constitute approximately 40% and 30% of the mitochondrial membranes, respectively [46]. Experimental choline deficiency in lower fat diet formulations than those tested here causes a decreased ratio of total hepatic PC/PE [44,47–49] and hepatic mitochondrial dysfunction [38–40]. Furthermore, NAFLD patients have a reduction of the total hepatic PC/PE ratio and impaired hepatocyte membrane integrity [44,50]. Maintenance of mitochondrial function requires homeostatic membrane phospholipid composition to coordinate surface charge, stereoelectronic relationships, and membrane dynamics that each effect membrane function and structural integrity, critical determinants of mitochondrial electron transport and oxidative phosphorylation [51–53].

**Figure 9:**
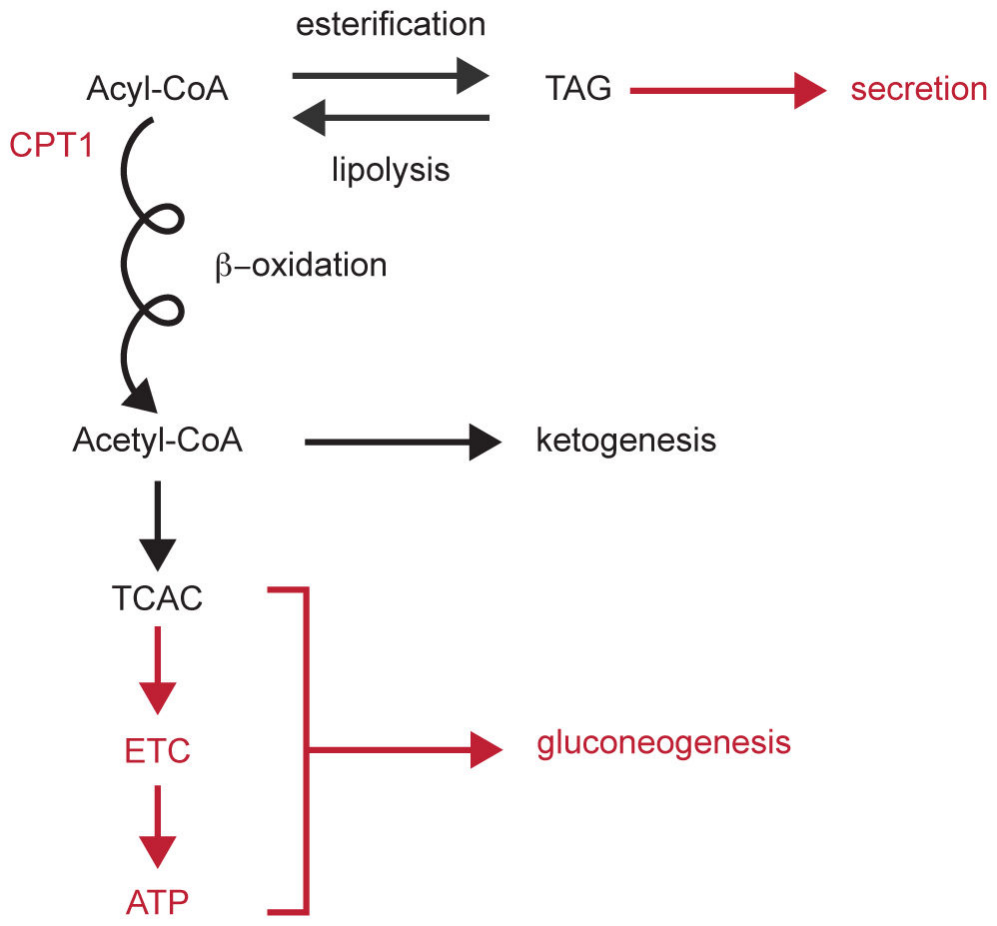
Influence of choline restriction in integrated hepatic mitochondrial fatty acyl-CoA metabolism. Red text and red arrows highlight hepatic processes known to be impaired by administration of experimental choline deficient diets. Unlike previous observations using lower fat choline deficient versus replete formulations, in this study, choline repletion in ~90% kcal fat diets did not alter triacylglycerol (TAG) secretion as VLDL. However, choline restriction in a ~90% kcal fat diet was associated with mitochondrial structural and functional abnormalities, which were linked to liver fat accumulation and injury. CPT1, carnitine palmitoyltransferase 1; TCAC, tricarboxylic acid cycle; ETC, electron transport chain; ATP, adenosine triphosphate.

Adaptation to a diet with ~90% kcal derived from fat requires integration of hepatic mitochondrial fatty acyl-CoA transport, esterification and storage in neutral pools, lipoprotein secretion, and lipolysis, with mitochondrial β-oxidation, ketogenesis, tricarboxylic acid (TCA) cycle, electron transport, and oxidative phosphorylation (Figure 9), and impairment of hepatic mitochondrial fatty acid oxidation contributes to liver fat accumulation [54]. Effective procession of fatty acids through integrated mitochondrial pathways also stimulates gluconeogenesis [55,56]. Of the four paste diets tested, however, only VLP/C^-^-fed mice were not hyperglycemic. Moreover, basal hepatic glucose production of mice maintained on the VLP/C^-^ parent diet Bio-Serv F3666 is normal, despite delivering > 90% kcal as fat, and a poor hepatic suppressive response to exogenous insulin [21]. A likely contributor to relative hypoglycemia in VLP/C^-^ and Bio-Serv F3666-fed mice is hepatic mitochondrial dysfunction that prevents adequately matched fatty acyl-CoA mitochondrial uptake and terminal oxidation. Differences in extrahepatic glucose disposal could also contribute to relative hypoglycemia in VLP/C^-^ mice, but glucose excursions among the four paste diets were not significantly different (data not shown). Hyperglycemia in mice fed LP/C^-^, despite steatosis and likely hepatic mitochondrial dysfunction, is multifactorial but is most likely attributable to additional dietary glucogenic amino acids that support gluconeogenesis after conversion to TCA cycle intermediates.

Choline deficient diets provoke defects in carnitine palmitoyltransferase (CPT 1) dependent fatty acyl-CoA transport [57], palmitate oxidation [57], electron transport [58], and as a result, gluconeogenesis [59,60] (Figure 9). Conversely, ketogenesis is not directly impaired by choline deficiency: both the fate-committing ketogenic enzyme, mitochondrial hydroxymethylglutaryl-CoA synthase (HMGCS2), and the enzyme catalyzing the subsequent ketogenic reaction, hydroxymethylglutaryl-CoA lyase, are soluble mitochondrial matrix enzymes that are influenced only indirectly by dysfunctional mitochondrial membrane structure and function. The results of these experiments strongly suggest that the robust ketosis in VLP/C^-^ mice reports the relative fraction of fatty acyl-CoAs that successfully complete hepatic β-oxidation, and not those that fail to gain entry into mitochondria or those that cannot complete terminal oxidation. Therefore, compared to their choline restricted counterparts, ketosis was less pronounced in mice fed the very high fat, choline replete diets in part because integrated hepatic mitochondrial function supports mitochondrial acyl-CoA uptake and terminal oxidation. Conversely, relative suppression of ketosis in mice fed choline restricted LP/C^-^, compared to mice fed VLP/C^-^, is attributable to (i) the sensitivity in rodents to amino acid-mediated insulin secretion [22,61], which in turn suppresses ketogenesis [62], and (ii) the modest ability of amino acid-derived gluconeogenic metabolites to suppress ketogenesis [63].

The independent contributions of choline and methionine deficiencies to abnormal hepatic metabolism and pathology in mice are incompletely understood because studies performed heretofore suggest macronutrient context-dependent effects of each of these micronutrients. In the context of carbohydrate-enriched diets, selective choline deficiency drives hepatic steatosis, while selective methionine deficiency has been linked to hepatic inflammation, oxidative stress and hepatocellular injury [64]. Conversely, mice fed a high fat (60% kcal), 26% kcal carbohydrate diet are protected against hepatic TAG accumulation, and do not exhibit overt inflammation or hepatocellular injury by methionine restriction [65]. Because thresholds for methionine deficiency-induced hepatocellular injury are unknown and are highly dependent on macronutrient distribution, these studies were not designed to definitively segregate the independent roles of methionine and choline restriction in the observed hepatic phenotypes. Nonetheless, this study supports the supplementation of choline in rodent experiments that are targeted to understand the physiological adaptations and responses to diet-induced ketosis. These experiments also demonstrate that nutritional induction of ketosis by a very high fat, very low carbohydrate diet in mice can occur in the context of maintained weight and hyperglycemia. Therefore, experimental use of VLP/C^+^ diet may allow mechanistic partitioning of the relative roles of glycemia versus ketosis in experimental conditions that are ameliorated by ketogenic diets.

An additional limitation of this study is that formal methods of hepatic biometry and stereology were not used for histopathological quantification of hepatocyte and fat droplet sizes [66], and for histopathology, relatively small numbers of animals were analyzed for each group. Nonetheless, hepatic histopathological characteristics were highly uniform among animals within each diet condition, and complementary formal biochemical quantification of hepatic fat was performed in larger numbers of animals. Moreover, effects of each of the five diets studied were determined in at least three separate cohorts of mice that were matched for age, strain, sex, and body weight at the time of initiation of each of the study diets. Analyses of histopathology, serum and hepatic biochemistry, and mitochondrial function were performed among these replicating cohorts to confirm reproducibility of the results. Future experiments will incorporate formal histopathological quantifications that reveal the mechanistic determinants relating these nutritional states to hepatic lipid droplet size and abundance.

In summary, these experiments demonstrate that the unique signature of hepatic lipid accumulation, inflammation, and cellular and mitochondrial injury induced in mice maintained on a very high fat, protein-restricted, very low carbohydrate, ketogenic diet can be linked to the adverse effects of choline deficiency on hepatic mitochondrial structure and function. The findings also underscore the concept that ketosis does not necessarily report effective hepatic fatty acid oxidation when supply overwhelms relative mitochondrial capacity. In setting of the VLP/C^-^ diet, ketogenesis provides a spill-over pathway that only partially compensates for inadequate fatty acid catabolism. However, it is important to note that while incompletely studied, ketogenic diets in humans are unlikely to cause mitochondrial dysfunction or fatty liver. Nonetheless, attentiveness to choline content, and its downstream effects on membrane phospholipid composition and function are important considerations when designing and measuring the physiological effects of low carbohydrate diets, which ultimately will require integration of biochemical approaches to quantify compartment-specific phospholipidomics with measurements of mitochondrial function, biogenesis, and autophagic turnover.
